# Draft Genome Sequence of *Clostridium* sp. Ne2, Clostridia from an Enrichment Culture Obtained from Australian Subterranean Termite, *Nasutitermes exitiosus*

**DOI:** 10.1128/genomeA.00304-15

**Published:** 2015-04-23

**Authors:** Han Wang, Hai Lin, Nai Tran-Dinh, Dongmei Li, Paul Greenfield, David J. Midgley

**Affiliations:** a University of Science and Technology, Beijing, China; b CSIRO, Food & Nutrition Flagship, Dickson, Australia; c Plant Health & Environment Laboratory, Ministry for Primary Industries, Auckland, New Zealand; d CSIRO, Digital Productivity and Services, Dickson, Australia; e CSIRO, Energy Flagship, Dickson, Australia

## Abstract

The draft genome sequence of *Clostridium* sp. Ne2 was reconstructed from a metagenome of a hydrogenogenic microbial consortium. The organism is most closely related to *Clostridium magnum* and is a strict anaerobe that is predicted to ferment a range of simple sugars.

## GENOME ANNOUNCEMENT

Biohydrogen is a promising energy source, being renewable, greenhouse neutral, and sourced from inexpensive feedstock (1). Hemicellulose is an attractive feedstock, being the second most abundant polysaccharide (2). Bacterial degradation of hemicellulose, and subsequent hydrogenogenesis via dark fermentation, requires an array of catabolic enzymes to degrade the hemicelluloses (3) along with hydrogenases (4, 5). A mixed-microbial culture, called 1 TC, from a worker *Nasutitermes exitiosus* (collected, 33°45′34″S; 150°59′25″E) demonstrated commercially significant hydrogen production at 30°C. The 1 TC consortia was almost exclusively composed of three clostridial taxa: one *Clostridium beijerinckii* strain (Ne1) (6), one *Ruminoclostridium* species (Ne3) (7), and, finally, the subject of this paper, Ne2, a taxon most closely related to, but likely distinct from, *Clostridium magnum*, *Clostridium carboxidivorans*, and *Clostridium ljungdahlii*.

*Clostridium* sp. Ne2 accounted for 25.2% of the metagenome. It was separated from the metagenome using short k-mer methods (8) and manual inspection. The draft genome for Ne2 included 283 large (>200 bp) contigs, totalling ~5.2 Mbp, with size distributions of 18,507 bp, 13,411 bp, and 23,721 bp for the mean, median, and *N*_50_ contig lengths. Annotation was performed using IMG-ER (Integrated Microbial Genomes Expert Review) (9), which predicted a total of 4,791 protein-coding genes and 38 structural RNAs. The annotated genome is available for download at IMG-ER (https://img.jgi.doe.gov/), and the sequences and metadata are available at the European Nucleotide Archive under accession no. PRJEB8629 (http://www.ebi.ac.uk/ena/data/view/PRJEB8629).

*Clostridium* sp. Ne2 is part of a clade that includes *C. magnum, C. ljungdahlii*, *C. carboxidivorans*, and *Tepidanaerobacter acetatoxydans* (JQ979073), although Ne2 is most closely related to *C. magnum*. The physiology of cultured organisms in this group includes homoacetogens and syntrophic acetate-oxidizing bacteria (10–14). It is likely that Ne2 is similar in its physiology, and if this is correct, it presumably can both ferment simple sugars to hydrogen and subsequently consume hydrogen in the absence of sugars. Pfam classification of genes (http://pfam.xfam.org/) (15) from the Ne2 genome suggests the presence of a number of iron (FeFe) and nickel iron (NiFe) hydrogenases (PF02906.9; PF02256.12; PF00374.14; PF14720.1). *Clostridium magnum* appears to require very small amounts of yeast extract (0.025%) for hydrogen metabolism (11), presumably as vitamins or other co-factors. It is worth noting, however, that no yeast extract was included in the medium used to grow the 1 TC consortia.

Growth on xylan as a sole source of carbon is facilitated by endo-acting xylanases, other xyloglucanase and xylosidases, though numerous accessory enzymes are required for complete digestion. Analysis using dbCAN (http://csbl.bmb.uga.edu/dbCAN/index.php) (16) suggests that *Clostridium* sp. Ne2 possesses xylanases (GH28, GH30), one probable xyloglucanase (GH74), and various enzymes which target hemicellulose-derived oligosaccharides and side branches (GH1, -3, -4, -39, -42, -43, and -127).

It is thus unclear what roles Ne2 performs within the 1 TC consortia. It may be that in the presence of xylan, Ne2 acts as a heterotroph, assisting with the degradation of xylan and, as this resource becomes depleted, switches to an autotrophic mode of physiology, consuming hydrogen as a homoacetogen. Further work is required to elucidate the role of this organism within the consortia.

### Nucleotide sequence accession numbers. 

This whole-genome shotgun project has been deposited at DDBJ/EMBL/GenBank under the accession numbers CEME01000001 through CEME01000283.
